# Resistance to oxyimino-cephalosporins conferred by an alternative mechanism of hydrolysis by the *Acinetobacter*-derived cephalosporinase-33 (ADC-33), a class C β-lactamase present in carbapenem-resistant *Acinetobacter baumannii* (CR*Ab*)

**DOI:** 10.1128/mbio.00287-25

**Published:** 2025-05-16

**Authors:** Rachel A. Powers, Bradley J. Wallar, Hannah R. Jarvis, Zoe X. Ziegler, Cynthia M. June, Christopher R. Bethel, Andrea M. Hujer, Magdalena A. Taracila, Susan D. Rudin, Kristine M. Hujer, Fabio Prati, Emilia Caselli, Robert A. Bonomo

**Affiliations:** 1 Department of Chemistry, Grand Valley State University 1142, Allendale, Michigan, USA; 2 Research Service, Louis Stokes Cleveland Department of Veterans Affairs Medical Center 465630, Cleveland, Ohio, USA; 3 Department of Medicine, Case Western Reserve University School of Medicine 220786, Cleveland, Ohio, USA; 4 Department of Life Sciences, University of Modena and Reggio Emilia 117706, Modena, Emilia-Romagna, Italy; 5 Louis Stokes Cleveland Department of Veterans Affairs Medical Center 20083, Cleveland, Ohio, USA; 6 Departments of Pharmacology, Molecular Biology and Microbiology, Biochemistry, and Proteomics and Bioinformatics, Case Western Reserve University School of Medicine, Cleveland, Ohio, USA; 7 CWRU-Cleveland VAMC Center for Antimicrobial Resistance and Epidemiology (Case VA CARES), Cleveland, Ohio, USA; MedImmune, Gaithersburg, Maryland, USA

**Keywords:** β-lactamase, cephalosporinase, cefiderocol, ceftazidime, *Acinetobacter*, ADC-33, *Acinetobacter-*derived cephalosporinase

## Abstract

Antimicrobial resistance in *Acinetobacter baumannii* is partly mediated by chromosomal class C β-lactamases, the *Acinetobacter*-derived cephalosporinases (ADCs). Recently, a growing number of emerging variants were described, expanding this threat. Consistent with other β-lactamases, one of the main areas of variance exists in the Ω-loop region near the site of cephalosporin binding. Interestingly, a common alanine duplication (Adup) is found in this region. Herein, we studied specific Adup variants expressed in a uniform *Escherichia coli* genetic background that demonstrated high-level resistance to multiple oxyimino-cephalosporins. For ceftolozane and ceftazidime, the Adup ADCs significantly increased levels of resistance (minimum inhibitory concentration [MIC] ≥ 512 µg/mL and MIC ≥ 1,024 µg/mL, respectively). These observations were consistent with the increased *k*_cat_/*K*_*M*_ for ceftazidime. For cefiderocol, three Adup variants exhibited increased MICs and increased *k*_cat_/*K*_*M*_ for this compound. Timed electrospray ionization mass spectrometry demonstrated stable cephalosporin:ADC adducts with ADC-30 (non-Adup), but not with ADC-33 (Adup), consistent with turnover. The X-ray crystal structure of Adup variant ADC-33 in complex with ceftazidime was determined (1.57 Å resolution) and suggests that increased turnover is facilitated by conformational changes (shift in Tyr221 and orientation of the oxyimino portion of the R1 side chain) and repositioning of water in the active site. These changes appear to favor substrate-assisted catalysis as an alternative mechanism to base-assisted catalysis. These studies also provide unprecedented insight into the mechanism underlying oxyimino-cephalosporin hydrolysis by expanded-spectrum ADC β-lactamases and possibly other class C β-lactamases, which is of critical importance to future drug design.

**IMPORTANCE:**

The characterization of emerging *Acinetobacter*-derived cephalosporinase (ADC) variants is necessary to understand the increasing resistance to β-lactam antibiotics in *Acinetobacter* spp. In this study, cefiderocol retains effectiveness against ADC variants with and without an Ω-loop alanine duplication (Adup). However, the presence of the Adup appears to introduce loop flexibility and structural alterations resulting in increased resistance and steady-state turnover of larger cephalosporins. Further characterization provides unprecedented insight into the mechanism of cephalosporin hydrolysis by ADC β-lactamases and supports a concomitant increase in ADC structural flexibility and cephalosporin affinity that leads to more efficient hydrolysis. In addition, the crystal structure of ADC-33 in complex with ceftazidime is consistent with a substrate-assisted catalysis mechanism. The structural differences in the ADC-33 active site leading to ceftazidime catalysis provide a better understanding of β-lactamase Adup variants and open important opportunities for future drug design and development.

## INTRODUCTION

Antimicrobial resistance is a major global health threat, and multidrug-resistant *Acinetobacter baumannii* (*Ab*), a gram-negative opportunistic pathogen, is responsible for a frightening number of hospital-acquired infections. *A. baumannii* is classified by the World Health Organization as a critical group pathogen (1), and the Centers for Disease Control and Prevention identified significant increases in infections across many healthcare-associated pathogens (2). As these isolates are often resistant to nearly all antibiotics, they present a continuous healthcare emergency.

Cephalosporin resistance in carbapenem-resistant *A. baumannii* (CR*Ab*) is largely mediated by *Acinetobacter*-derived cephalosporinases (ADCs), class C β-lactamases present in all *Acinetobacter* spp. (3). Most class C β-lactamases, including ADCs, hydrolyze amino- and ureidopenicillins and cephamycins. In contrast, oxyimino-cephalosporins, such as ceftazidime (CAZ) and cefotaxime, are hydrolyzed less efficiently. Previous studies in our lab demonstrated that ADC-7 is an efficient narrow-spectrum cephalosporinase (3). The hydrolysis of cephaloridine and cephalothin approached the upper limits of catalytic efficiency of any class C β-lactamase, yet the ability to efficiently hydrolyze CAZ or cefepime was lacking. In fact, CAZ inhibits ADC-7 with low micromolar affinity, supported by the capture of the crystal structure of the ADC-7/CAZ acyl-enzyme complex (4). In contrast, other ADCs (e.g., ADC-33 and ADC-56) exhibit the ability to hydrolyze expanded-spectrum cephalosporins (e.g., CAZ, cefotaxime, and cefepime) (5–7). The emergence of these expanded-spectrum AmpCs (ESACs) in the clinic with the ability to hydrolyze all cephalosporins is particularly worrisome as a major thrust in pharmaceutical development is to optimize this class for combination with novel β-lactamase inhibitors (cefepime/taniborbactam and cefiderocol/xeruborbactam) and as carbapenem-sparing agents (cefepime/enmetazobactam).

**Fig 1:**
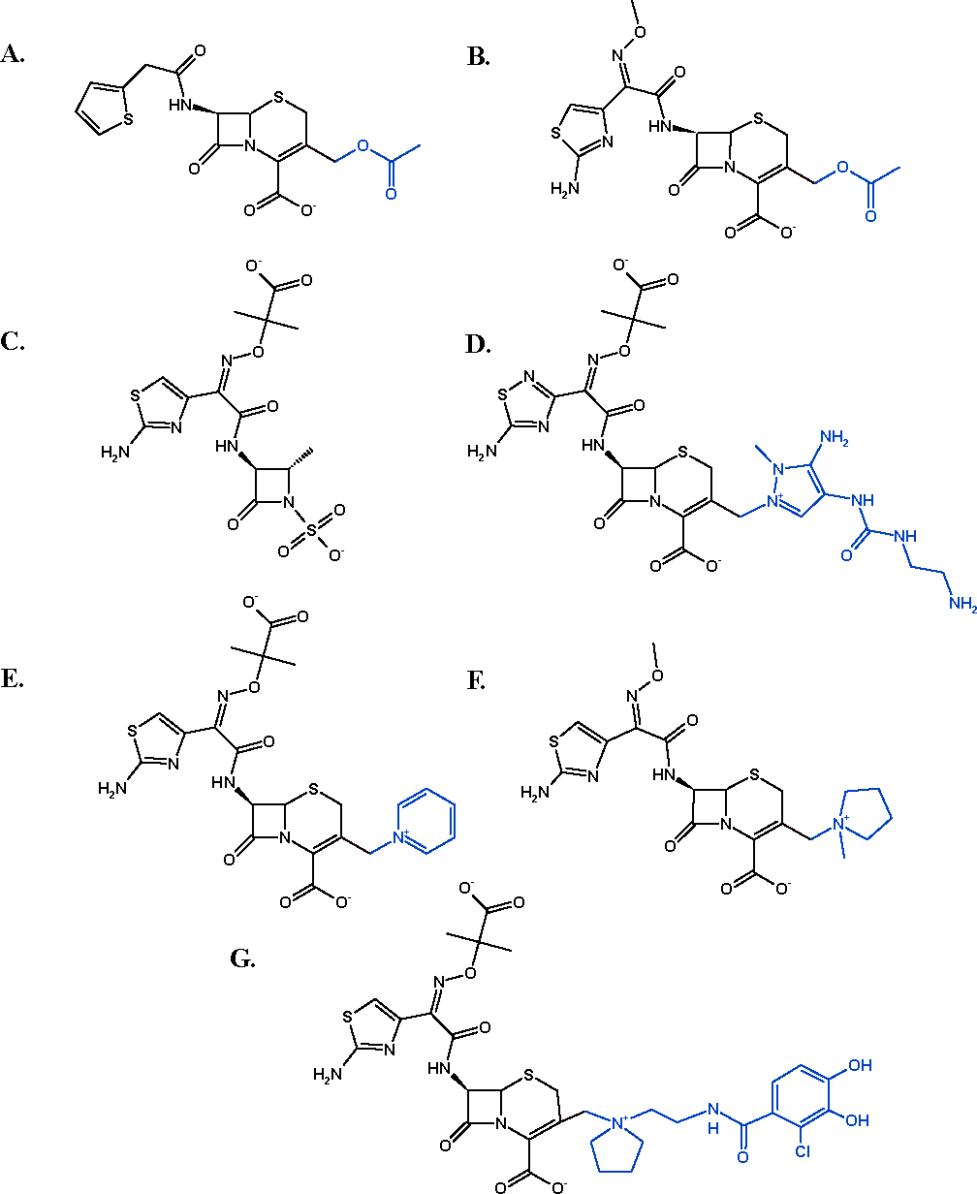
Structures of antibiotic substrates used in this study: (A) cephalothin; (B) cefotaxime; (C) aztreonam; (D) ceftolozane; (E) CAZ; (F) cefepime; (G) FDC. The R2 group is shown in blue.

Reduced susceptibility to CAZ is demonstrated in bacteria harboring ESACs, and many confer resistance to cefepime (6–8). As compared to earlier cephalosporins, like cephalothin (Fig. 1A), newer cephalosporins are characterized by a larger overall size, possessing similar R1 groups (e.g., cefotaxime, aztreonam, ceftolozane, CAZ, cefepime, and cefiderocol [FDC]; Fig. 1B through G). Additionally, some of the compounds contain a positive charge in their R2 leaving groups (CAZ, cefepime, and FDC; Fig. 1E through G).

ESACs usually possess amino acid substitutions or insertions/deletions in four regions of the enzyme that are located near or at the active site: Ω-loop, H-10 helix, H-2 helix, and C-terminus (6, 9, 10). Representative ESACs include GC1 in *Enterobacter*, CMY-30, -32, and -33 in *Citrobacter*, and ADC-33, -53, and -56 in *Acinetobacter* (11). In *Pseudomonas*, certain *Pseudomonas*-derived cephalosporinases (PDCs; PDC-85, -86, and -221) have substitutions (e.g., Glu219Lys and Tyr221His) that enable increased resistance to the newest cephalosporins (11–16). These PDCs show increased activity against ceftolozane and CAZ. However, the exact mechanism of the ESAC phenotype remains unknown.

We previously demonstrated that several ADC variants with an alanine duplication (Adup) in the Ω-loop gained the ability to hydrolyze both CAZ and FDC (5). We sought to examine the mechanism by which these cephalosporins are hydrolyzed by this family of class C enzymes to provide increased resistance. We used a combination of microbiology, mass spectrometry, enzyme kinetics, molecular modeling, and X-ray crystallography to investigate the changes in the ADC active site of specific variants that cause the observed increase in resistance. Our investigations uncovered an alternative pathway to the hydrolysis of oxyimino-cephalosporins in ADC that has significant implications for future medicinal chemistry efforts.

## RESULTS

### Microbiological antibiotic susceptibility of ADC variants

**Fig 2:**
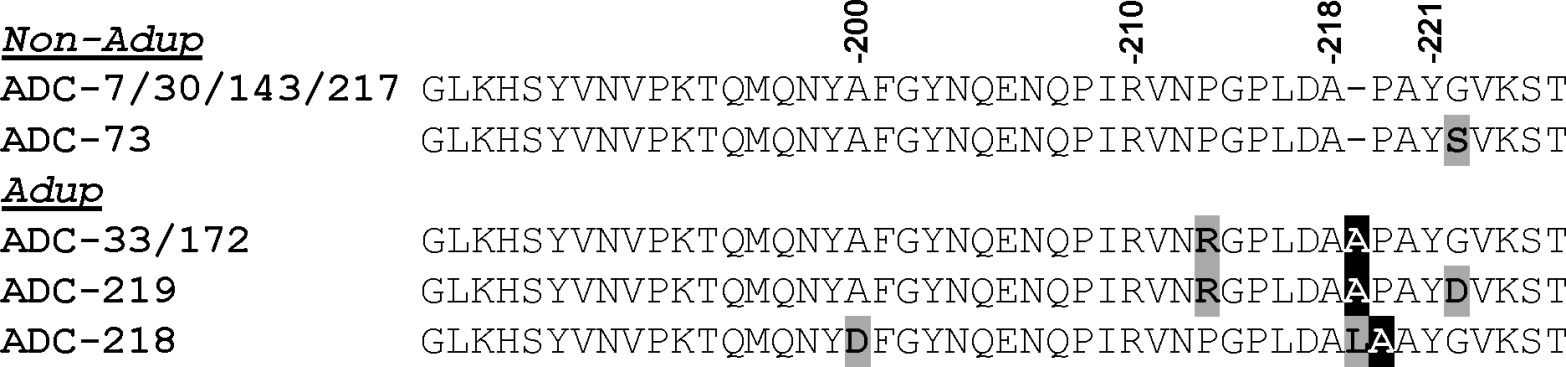
Multiple alignment of the Ω-loop region of ADC variants with or without an Adup using Clustal Omega (18). Other variations are bolded and shaded in gray. The structural alignment-based numbering of class C β-lactamases (SANC) scheme is used to label amino acid residues. The specific mutations not shown above are single substitutions of ADC-30: ADC-143 (ADC-30 Q120K) and ADC-217 (ADC-30 V262E). The GenBank reference sequence for each ADC variant is as follows: ADC-7 (WP_063857816.1), ADC-30 (WP_001211218.1), ADC-33 (WP_001211220.1), ADC-73 (WP_001211219.1), ADC-143 (WP_033502167.1), ADC-172 (WP_032027415.1), ADC-217 (WP_060470109.1), ADC-218 (WP_078216190.1), ADC-219 (WP_039213353.1).

An expanded set of ADCs was selected to build upon our previously reported elevated minimum inhibitory concentrations (MICs) toward CAZ and FDC for variants containing an Adup in the Ω-loop (Fig. 2). It should be noted that Adup is used as a way to distinguish variants that contain at least an alanine duplication in the Ω-loop with one or more variations in the sequence. ADC-33 was previously reported to demonstrate hydrolytic activity against CAZ and FDC (*k*_cat_ 3.71 and 0.60 s^−1^, respectively [5]). The expanded set of *Ab* ESAC enzymes containing an alanine duplication in the Ω-loop (Adup: ADC-33, -172, -218, -219) was compared to variants without the duplication (non-Adup: ADC-30, -73, -143, -217). Importantly, ADC-73 and -30 are two of the most common ADC variants in *Ab*. The prevalence of the ADC variants included in this study, from an analysis of 28,330 NCBI deposited *Ab* genomes, is as follows: ADC-73 (28.10%), ADC-30 (25.15%), ADC-33 (3.46%), ADC-219 (0.12%), ADC-143 (0.1%), ADC-217 (0.08%), ADC-172 (0.01%), ADC-218 (0.01%), and ADC-7 (0%) (17). Using a panel of β-lactam antibiotics, MICs were determined for the ADC isolates to assess differences between Adup and non-Adup variants (Table 1).

**TABLE 1:** MIC results in µg/mL for the ADC variants in DH10B *Escherichia coli*

ADC variant	Adup	CEF^*a*^ (µg/mL)	CTX (µg/mL)	ATM (µg/mL)	TOL (µg/mL)	CAZ (µg/mL)	FDC (µg/mL)	FEP (µg/mL)	IPM (µg/mL)
ADC-7	No	4096	64	8	8	64	0.06	0.5	0.25
ADC-30	No	4096	32	8	4	64	0.125	0.5	0.25
ADC-73	No	2048	32	2	2	64	0.125	0.25	0.25
ADC-143	No	2048	32	16	16	256	0.125	0.25	0.25
ADC-217	No	2048	64	8	16	128	0.25	16	0.25
ADC-33	Yes	2048	64	64	512	1024	0.5	1	0.25
ADC-172	Yes	2048	32	32	512	1024	1.0	8	0.25
ADC-218	Yes	2048	256	256	512	2048	0.5	4	0.25
ADC-219	Yes	1024	32	32	128	64	0.125	0.125	0.25
pBCSK− no insert	NA	8	0.06	0.12	0.5	0.25	0.125	0.03	0.25
DH10B alone	NA	16	0.12	0.25	0.5	0.5	0.06	0.06	0.25

^*a*^ Abbreviations of antibiotics used: ATM, aztreonam; CEF, cephalothin; CTX, cefotaxime; FEP, cefepime; IPM, imipenem; TOL, ceftolozane.

Adup variants in DH10B did not show a significant increase in MICs for cephalothin and cefotaxime, yet a significant gain in resistance to antibiotics containing similar bulky R1 groups was observed (aztreonam, ceftolozane, and CAZ; Fig. 1). Although with the monobactam aztreonam, we observed a modest increase in resistance (ADC-30/-33; 8 µg/mL/64 µg/mL), Adup ADCs provided a significant increase to ceftolozane (ADC-30/-33; 4 µg/mL/512 µg/mL) and CAZ (ADC-30/-33; 64 µg/mL/1,024 µg/mL). However, Adup ADC-219 did not demonstrate an increase in resistance to CAZ.

Adup variants ADC-33/-172/-218 provide a modest increase in resistance to cefepime, up to a 16-fold increase from ADC-30 to ADC-172 (Table 1). Interestingly, non-Adup ADC-217 has the highest cefepime resistance (16 µg/mL).

FDC is largely spared from the gain in resistance to cephalosporins, such as ceftolozane and CAZ. ADC-172 provided the highest MIC against FDC (1 µg/mL), yet all studied ADCs remained in the susceptible range. Both ceftolozane and CAZ contain an R1 group similar to FDC, with the R2 group being the main difference (Fig. 1). The large increase in resistance to CAZ versus FDC by the Adup variants provided a distinct boundary of resistance to β-lactam antibiotics. Therefore, binding and turnover of CAZ and FDC by the ADCs were further characterized. None of the ADCs provided resistance to the carbapenem imipenem (0.25 µg/mL). ADC variant protein expression levels were qualitatively assayed by immunoblot analysis (Fig. S1). The analysis was not quantitative as two antibody reactive epitopes were found in the Ω-loop upon epitope mapping of ADC-7 using the anti-ADC-7 rabbit polyclonal antibody (detailed explanation in Fig. S1). All ADC variants were expressed and detected by immunoblot analysis.

### Kinetic analysis of ADC variants with CAZ and FDC

Adup variant ADC-33 efficiently hydrolyzes CAZ (5, 6), whereas most ADCs cannot. Previous studies have shown that CAZ acts as an inhibitor of the non-Adup ADC-7 (3). Using steady-state kinetics, we explored *K*_*i*_ values of CAZ and FDC to all ADC variants, as well as their ability to hydrolyze both antibiotics (Table 2).

**TABLE 2:** Kinetic analysis for FDC and CAZ for the ADC variants

ADC variant	Adup	CAZ *k*_cat_/*K*_*M*_ (μM^−1^s^−1^)	FDC *k*_cat/_*K*_*M*_ (μM^−1^s^−1^)	CAZ *K*_*i*_^*d*^ (μM)	FDC *K*_*i*_^*d*^ (μM)
ADC-7	No	*0.0020^a^^,^^b^*	*<0.001* ^*a*^	2.10 ± 0.25	75.0 ± 5.32
ADC-30	No	*0.0033^a^*	*<0.001* ^*a*^	3.23 ± 0.31	62.7 ± 3.95
ADC-73	No	*0.0026*	*<0.001*	8.72 ± 0.74	110.7 ± 9.70
ADC-143	No	*0.0047*	*<0.001*	47.8 ± 3.34	171.3 ± 27.7
ADC-217	No	*0.0025*	*<0.001*	0.26 ± 0.014	0.21 ± 0.025
ADC-33	Yes	0.0626 ± 0.0083*^a,c^*	0.0056 ± 0.0007*^a^^,c^*	28.4 ± 2.09	19.3 ± 1.46
ADC-172	Yes	0.0857 ± 0.0075^*c*^	0.016 ± 0.0012^*c*^	12.7 ± 1.45	3.26 ± 0.31
ADC-218	Yes	*0.0300*	*<0.001*	490.6 ± 45.8	615.6 ± 131.0
ADC-219	Yes	*0.015* ^*a*^	*0.001* ^*a*^	>1,000	>1,000

^*a*^ Values were previously reported in reference 5 and are provided here for comparison.

^*b*^ All *k*_cat_*/K*_*M*_ values in italics were calculated from linear fits of *v*_o_ in low [S] ranges (<0.08 * *K*_*M*_). The standard errors for these *k*_cat_*/K*_*M*_ values were lower than 5%.

^*c*^ The *k*_cat_ and *K*_*M*_ values that could be individually measured (i.e., ADC-33 and ADC-172) are as follows. ADC-33 with CAZ: *k*_cat_, 3.71 ± 0.088 s^−1^; *K*_*M*_, 59.3 ± 7.80 µM. ADC-33 with FDC: *k*_cat_, 0.600 ± 0.0315 s^−1^; *K*_*M*_, 107.2 ± 13.8 µM. ADC-172 with CAZ: *k*_cat_, 2.30 ± 0.056 s^−1^; *K*_*M*_, 26.9 ± 2.35 µM. ADC-172 with FDC: *k*_cat_, 0.348 ± 0.0066 s^−1^; *K*_*M*_, 22.2 ± 1.74 µM. The corresponding Michaelis-Menten plots are shown in Fig. S2.

^*d*^ While it is likely that these *K*_*i*_ values represent binding affinities for CAZ and FDC, it is possible that effects on catalytic rate constants also contribute to the observed differences.

Steady-state/competition kinetics demonstrated that non-Adup ADC-217 had the lowest *K*_*i*_ values for both CAZ and FDC (0.26 and 0.21 µM, respectively) but did not significantly turn over these antibiotics. The other non-Adups also did not hydrolyze CAZ and FDC and exhibited low micromolar *K*_*i*_ values for CAZ and an increased *K*_i_ value for FDC (ADC-30: *K*_*i*_^CAZ^ 3.23 µM, *K*_*i*_^FDC^ 62.7 µM). Adup variants ADC-33 and ADC-172 exhibited low micromolar *K*_*i*_ values for both CAZ and FDC and no significant increase in *K*_*i*_ for FDC, whereas ADC-218 and ADC-219 demonstrated much higher *K*_*i*_ values for both CAZ and FDC (ADC-218: *K*_*i*_^CAZ^ 491 µM, *K*_*i*_^FDC^ 616 µM).

Adup variants catalytically turn over larger cephalosporins, such as CAZ, with an approximate 10- to 26-fold increase in *k*_cat_/*K*_*M*_ over that of ADC-30 (0.0033 µM^−1^s^−1^), ranging from 0.0300 (ADC-218) to 0.0857 µM^−1^s^−1^ (ADC-172). In addition to the previously determined values for ADC-33 (*k*_cat_^CAZ^ 3.71 s^−1^, *K*_*M*_^CAZ^ 59.3 µM and *k*_cat_^FDC^ 0.60 s^−1^, *K*_*M*_^FDC^ 107.2 µM) (5), individual *k*_cat_ and *K*_*M*_ values for ADC-172 demonstrated similar turnover rates and *K*_*M*_ values (*k*_cat_^CAZ^ 2.30 s^−1^, *K*_*M*_^CAZ^ 26.9 µM and *k*_cat_^FDC^ 0.35 s^−1^, *K*_*M*_^FDC^ 22.2 µM). Based upon *K*_*i*_ values, ADC-33 and ADC-172 had similar binding affinities for CAZ and FDC, yet both had higher catalytic efficiencies for CAZ. The *k*_cat_/*K*_*M*_^CAZ^ of ADC-33 was ~10-fold higher (0.0626 versus 0.0056 µM^−1^s^−1^ for FDC).

### Mass spectrometry analysis of acylated adducts with ADC variants

**Fig 3:**
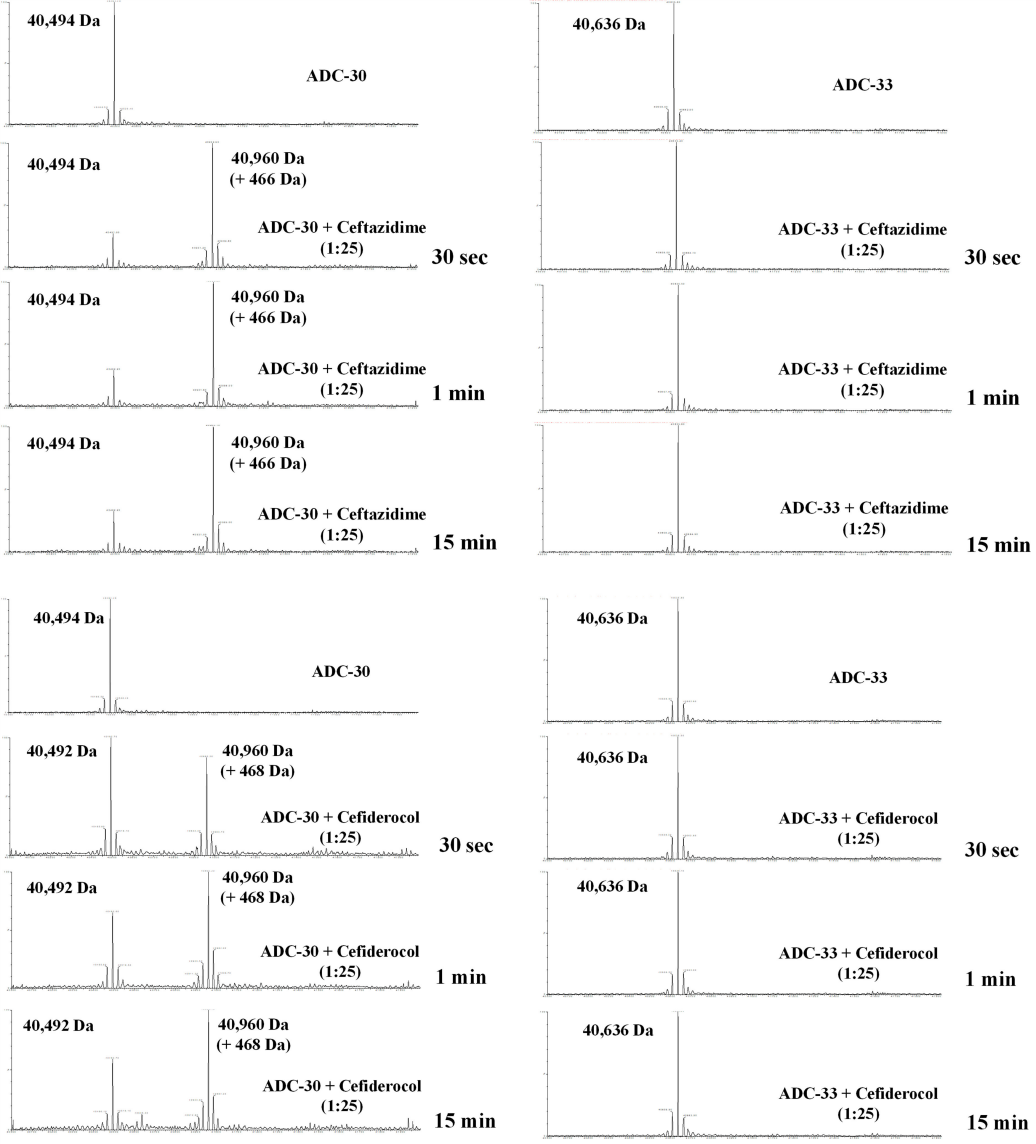
Electrospray mass spectrometry results for ADC-30 (non-Adup) and ADC-33 (Adup) incubated with CAZ and FDC for 0, 30 s, 1 min, and 15 min. The *x*-axis is mass (measured in daltons) and the *y*-axis is the intensity (0%–100%) of the signal for each mass.

The ability of non-Adup (ADC-30) and Adup (ADC-33) to form an acylated adduct with CAZ and FDC was investigated using timed electrospray ionization mass spectrometry (*t*-ESI-MS). ADC-33 did not form a stable acyl-enzyme adduct during incubation with either CAZ or FDC (30 s–1 h), consistent with deacylation and turnover of the substrates (Fig. 3). In contrast, for ADC-30, a stable +468 Da adduct was formed for ≤1 h (five time points collected) for both FDC and CAZ, although the percentage of acylated adduct varies from 70% for FDC to 20% for CAZ at 1 h. Overall, these data suggest that CAZ and FDC may serve as inhibitors of ADCs that do not contain a Ω-loop Adup and indicate the elimination of the R2 side chain upon acylation.

### Thermal denaturation of ADC variants

**Fig 4:**
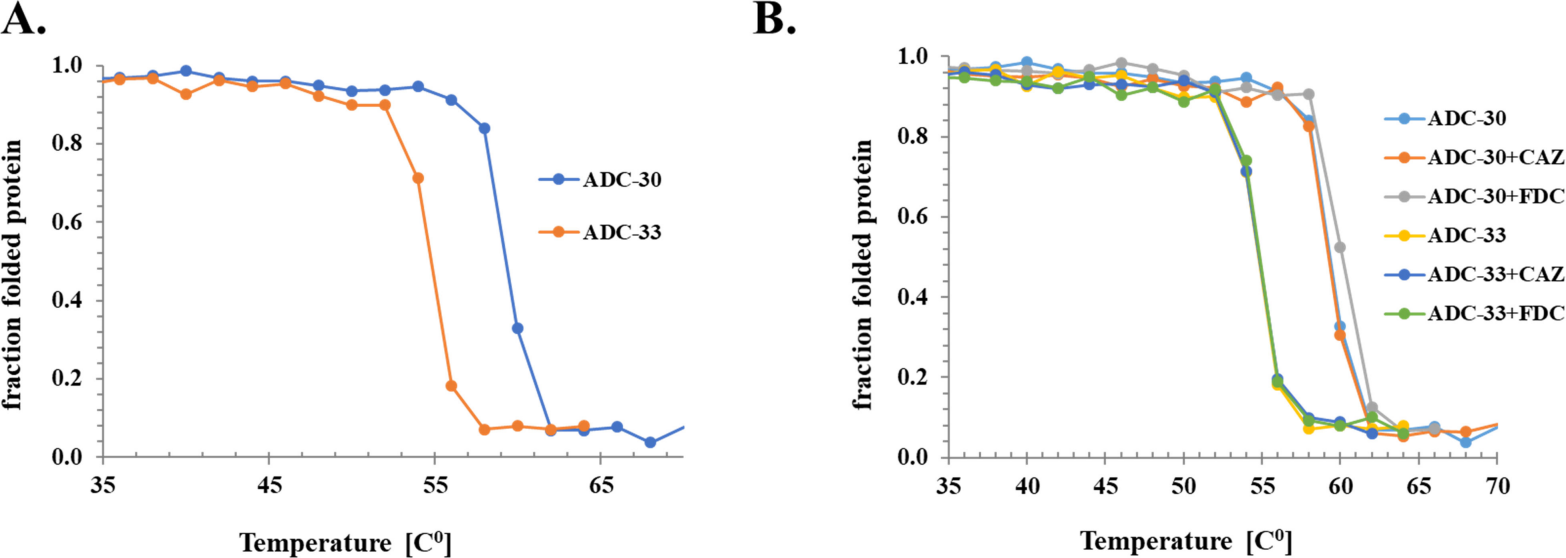
Thermal denaturation and melting curves for (**A**) ADC-30 and ADC-33 apo enzymes and (**B**) ADC-30 and ADC-33 with CAZ and FDC.

To explore overall β-lactamase stability, thermal denaturation experiments were performed with a representative non-Adup (ADC-30) and Adup variant (ADC-33). ADC-30 has a higher melting temperature (*T*_*m*_) than ADC-33 (*T*_*m*_^ADC-30^ 60°C; *T*_*m*_^ADC-33^ 55°C), suggesting decreased stability in ADC-33 due to the Adup (Fig. 4A).

Addition of CAZ does not alter the *T*_*m*_ of either β-lactamase, but FDC modestly stabilizes ADC-30 (*T*_*m*_ increases 2°C; Fig. 4B). The FDC-induced stabilization was not observed for ADC-33, consistent with the longer-lived ADC-30 adduct demonstrated by *t*-ESI-MS (Fig. 3).

### Structural analysis of ADC-33 in complex with CAZ

X-ray crystallography and molecular modeling were used to determine the structural basis and possible mechanism for CAZ hydrolysis by ADC-33. Might enhanced active site flexibility facilitate substrate binding and/or rearrangements necessary for catalysis? Based on sequence alignments (Fig. 2), flexibility in the Ω-loop could be introduced by the alanine duplication or a Pro213Arg present in three Adup variants (ADC-33, -172, and -219).

The X-ray crystallographic structure of ADC-33 in complex with CAZ was determined to 1.57 Å resolution (Table S1). ADC-33 crystallizes with two monomers in the asymmetric unit. Initial difference electron density maps (contoured at 3 σ) indicated the presence of CAZ in both monomers. In the B monomer, electron density was continuous with the Oγ atom of the catalytic residue Ser64, indicative of a covalent bond in an acyl-enzyme complex. In the A monomer, contiguous electron density to Ser64 was not observed, suggesting either a pre-covalent Michaelis-Menten or post-covalent product complex was captured. Both forms of CAZ were modeled into the A monomer. Electron density maps were consistent with the presence of the product form of CAZ (residue name A1BIM).

After refinement, residual difference electron density (contoured at 3 σ) remained in the B monomer, suggesting an additional form of CAZ in the active site. The product of CAZ (A1BIM) was added as an alternate conformation to the acyl-CAZ in the B monomer. The overall conformation of the product in each active site is maintained between the monomers. Electron density for the R1 dimethylcarboxylate group of both acylated and product forms of CAZ was not observed (Fig. S3). These atoms are not included in the final model.

**Fig 5:**
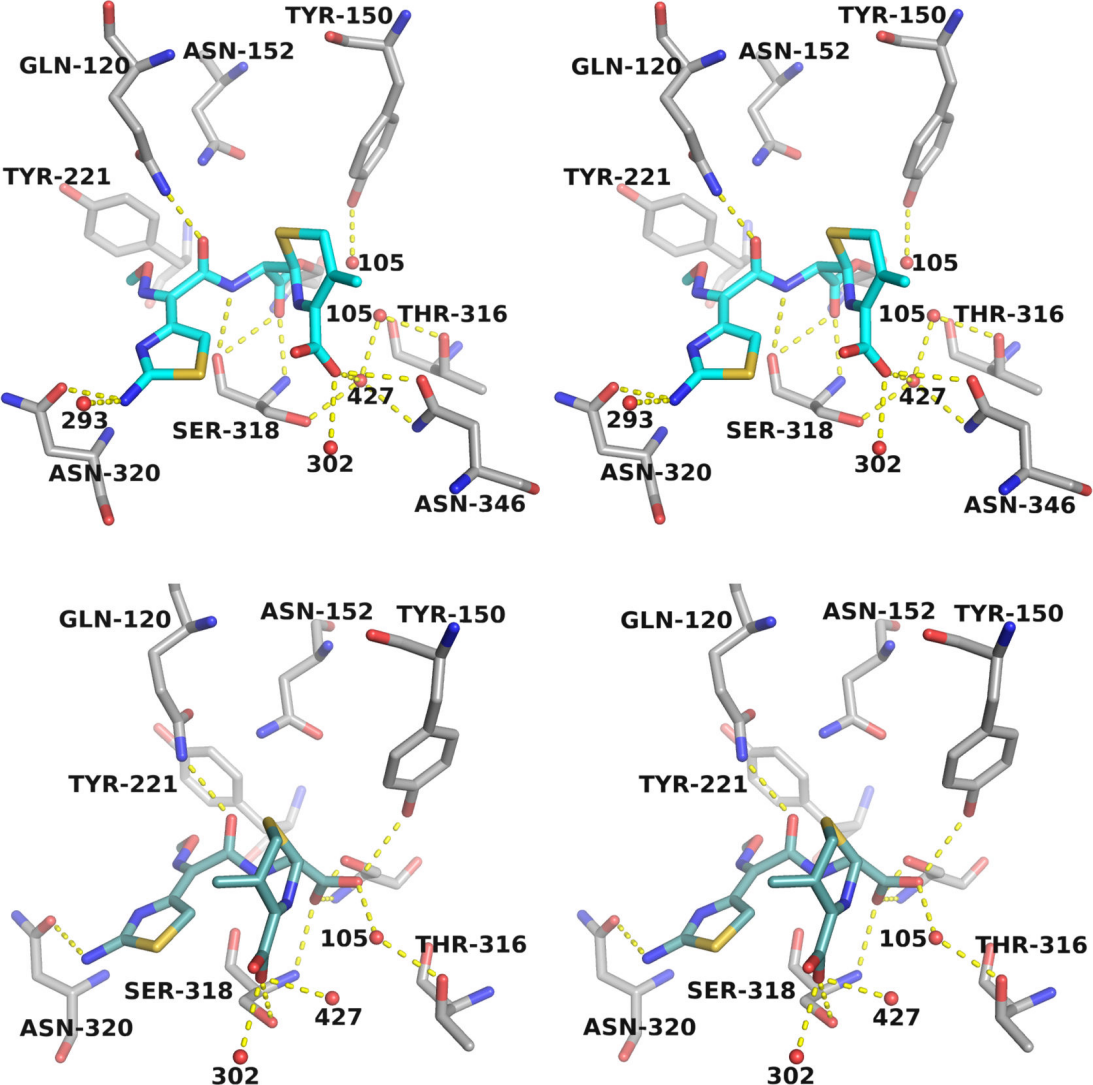
Stereoview of the hydrogen-bonding interactions in the ADC-33 complex with (top) acylated ceftazidime (CAZ, carbons cyan) and (bottom) product CAZ (A1BIM, carbons dark teal). Hydrogen bonds are indicated by yellow dashed lines and represent distances ~2.5–3.2 Å. ADC-33 protein carbon atoms are colored gray, nitrogens are dark blue, oxygens are red, and sulfurs are yellow.

The acyl and product versions of CAZ maintain interactions with key active site residues (Fig. 5). The oxyanion hole is occupied by an oxygen in both (carbonyl oxygen [O9] of acyl-CAZ and one carboxylate oxygen [O29] formed upon deacylation in product A1BIM), with hydrogen bonds formed with main chain amides (NH) of Ser64 and Ser318. Additionally, O9 of acyl-CAZ forms a close contact (2.9 Å) with the carbonyl oxygen of Ser318, presumably facilitating efficient deacylation in ADC-33. The carbonyl oxygen of the conserved R1 amide group maintains a hydrogen bond with Gln120 (Nε2) in both forms of CAZ, but the interaction with Asn152 (Nδ2, 3.4–3.7 Å) is not observed. In acyl-CAZ, the R1 amide NH maintains a hydrogen bond with the main chain carbonyl oxygen of Ser318. Finally, the C4′ carboxylate group of both interacts with the side chain of Ser318Oγ and two waters (Wat302 and Wat427), and in acyl-CAZ, is also within hydrogen-bonding distance to the side chain of Asn346.

Unique interactions between the acyl and product forms of CAZ are observed. In the product (A1BIM), one of the oxygens of the carboxylate formed upon deacylation (O28, derived from the deacylating water) hydrogen bonds with the side chain hydroxyl of Tyr150. Additionally, O28 hydrogen bonds with water molecule Wat105B and suggests a feasible approach for attack by the deacylating water. An alternate conformation of Wat105 (Wat105C) was modeled into the active site and would be present in the acylated (or apo) version. This water is ~2.9 Å from its electrophilic target, the carbonyl carbon of acyl-CAZ, as well as 2.8 Å from Tyr150OH. The nitrogen of the former lactam ring (N5) is ~3.4 Å from this water.

**Fig 6:**
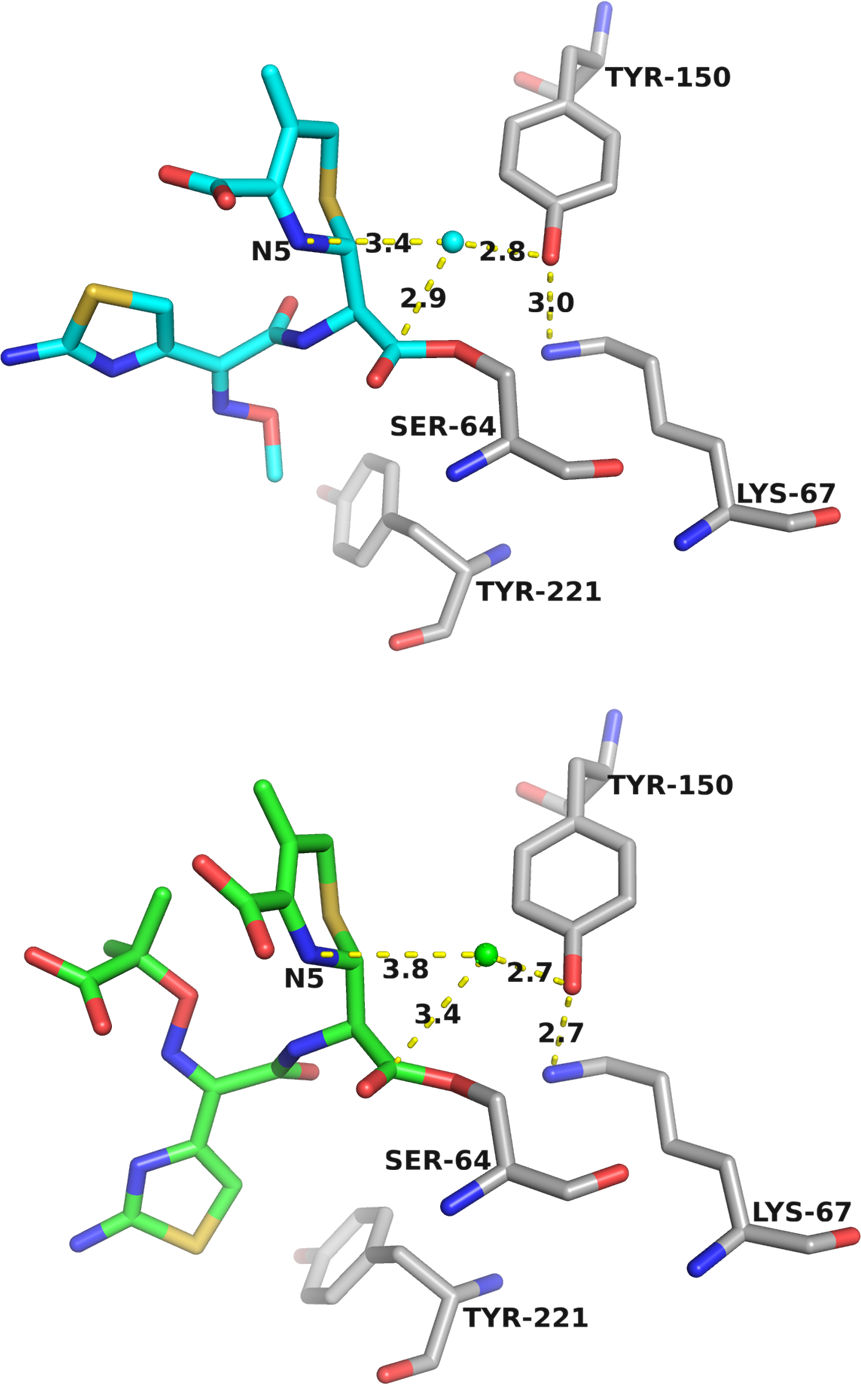
Comparison of ADC complexes with CAZ. In the ADC-33 complex with CAZ (cyan; top), the presumed deacylating water is indicated by the cyan sphere. The water is positioned appropriately for activation by the Tyr150:OH atom and ideally positioned for attack at the acyl carbonyl carbon (2.9 Å). This is different from what is observed in the complex of ADC-7 with CAZ (green; bottom), where the deacylating water is farther from N5 and the acyl carbonyl carbon, but is within hydrogen-bonding distance to Tyr150:OH.

**Fig 7:**
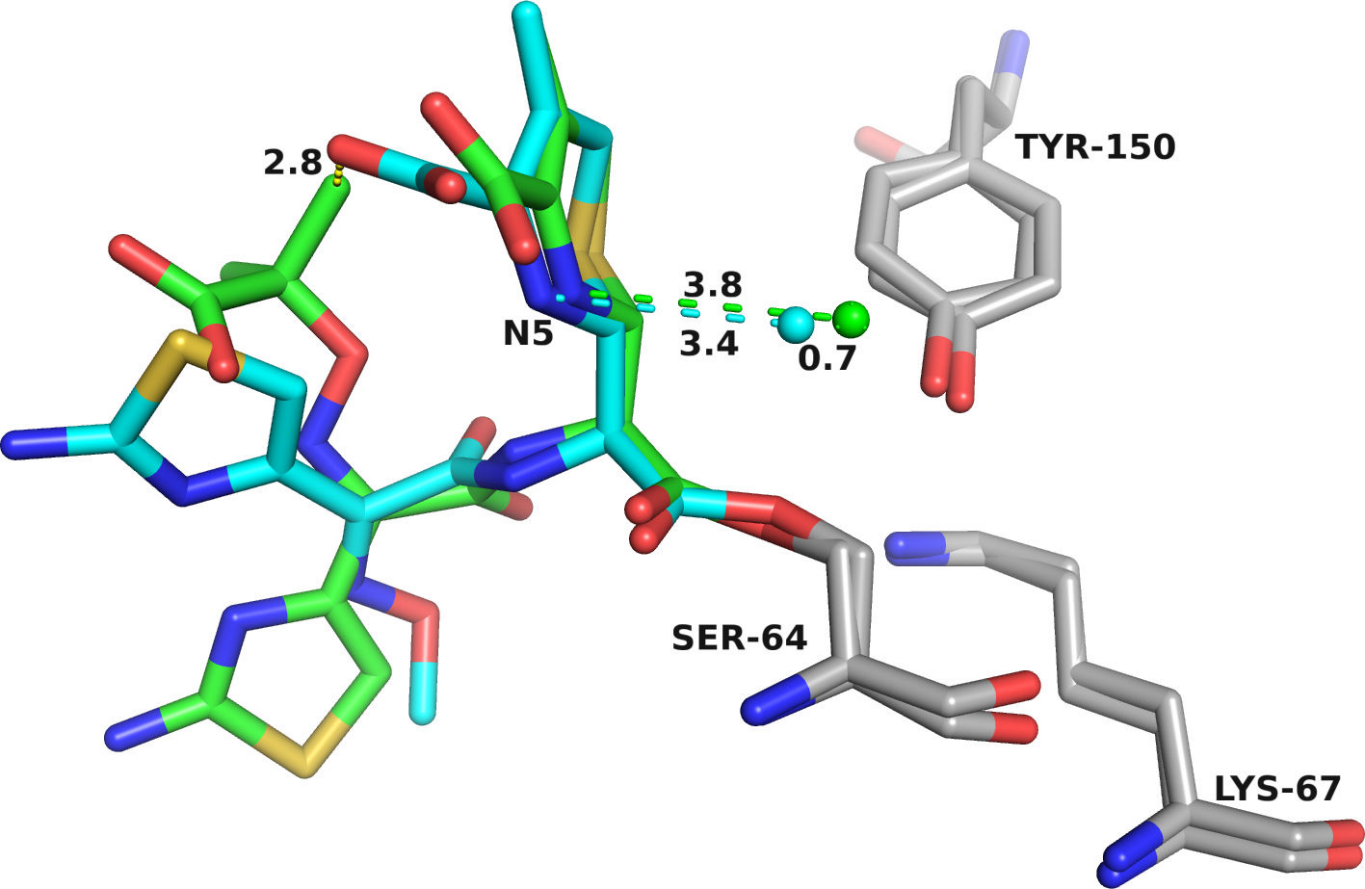
Superposition of ADC complexes with CAZ: ADC-7 (green) and ADC-33 (cyan). In ADC-33, the deacylating water is shifted 0.7 Å, positioning it closer to the carbonyl carbon (2.9 versus 3.4 Å), as well as closer to N5 (3.4 vs 3.8 Å) as compared to the ADC-7 complex. The unusual R1 side chain conformation in ADC-33 appears to facilitate this shift. The C4′ carboxylate adopts a different conformation that would clash with the oxyimino group (2.8 Å) if it adopted the one observed in the ADC-7/CAZ complex. The unusual R1 side chain conformation may only be possible because of the increased size/flexibility of the active site due to the Ala duplication in the Ω-loop.

Interestingly, the ADC-33 complex revealed that the oxyimino/aminothiazole portion of the R1 side chain of CAZ binds differently in the active site compared to the acyl-enzyme complex of ADC-7/CAZ (6PWM) (Fig. 6 and 7). Analogous to ADC-30, ADC-7 is a non-Adup and has a similar kinetic profile for β-lactam antibiotics (5). In the ADC-33 complex, the oxyimino portion of the R1 side chain is oriented toward Tyr221. This unusual conformation suggests a possible explanation for hydrolysis and product release. In the ADC-7/CAZ acyl-enzyme complex, a favorable stacking interaction is observed between Tyr221 and the aminothiazole ring of CAZ. In the ADC-33/product complex, electron density for the R1 side chain dimethylcarboxylate group is not observed, due to its presumed clash with Tyr221. Superposition of the complexes shows a shift of Tyr221 in the product complex that potentially favors product release. Indeed, despite several potential stabilizing interactions, a clash is observed between the carboxylate of the CAZ product and the catalytic Ser64 side chain. The carboxylate carbon atom (i.e., the former carbonyl carbon of the β-lactam ring) is within 2.6 Å of Ser64Oγ.

Multiple attempts to capture an ADC-33/FDC complex by soaking or co-crystallization were unsuccessful. Molecular modeling of ADC-33 with both cephalosporins docked into the active site was performed to explain the functional differences between CAZ and FDC. Initially, we used molecular docking to generate Michaelis-Menten complexes for both compounds starting with conformations that placed the carbonyl oxygen in the oxyanion hole of ADC-33 (Fig. S4). Based on resulting interaction energies, the best conformations show slightly different positioning of the two compounds in the active site. The larger FDC can be accommodated in the active site, although the conformation is more restricted, due to the presence of the bulky R2 catechol moiety in the Michaelis-Menten form.

Upon acylation, rearrangement of the β-lactam results in elimination of the unique R2 groups of both CAZ and FDC, producing indistinguishable acyl-enzyme intermediates at the molecular level. However, interestingly, the modeling suggests subtle differences that might explain mechanistic differences observed between CAZ and FDC.

**Fig 8:**
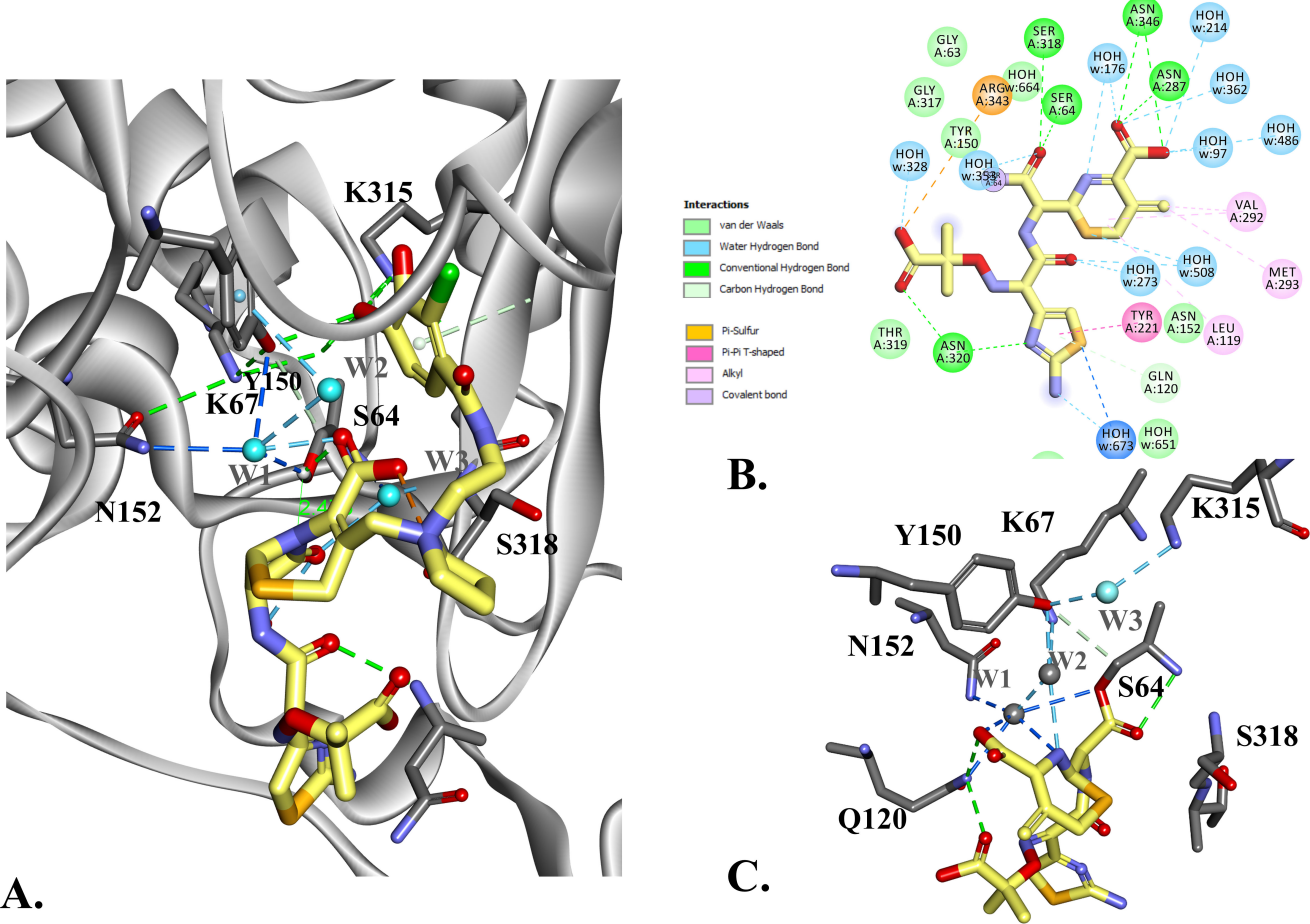
Molecular docking of ADC-33 with FDC as Michaelis-Menten (**A**), the initial conformation of the acylated FDC (**B**), and acyl-enzyme complex (**C**). Molecular modeling and MDS of the acylated FDC-ADC-33 complex suggest that FDC can adopt a different conformation into the active site of ADC-33. The initial conformation of the acylated FDC (**C**) shows multiple water molecules positioned at hydrogen-bond distances. After the MDS (**C**), two water molecules are recruited and positioned at hydrogen-bond distance: (W1) N152 and N5 of FDC dihydrothiazine ring; (W2) K67, Y150, N5, and C8 of FDC, and a third one (W3) coordinated by Y150 and K315. This suggests that the water molecules can be activated with the help from N5 of the FDC and is consistent with a substrate-assisted catalysis mechanism.

The acylation mechanism involves nucleophilic attack of the conserved Ser64 on the C9 carbonyl carbon of the β-lactam ring, forming a covalent acyl-enzyme intermediate. The molecular docking of FDC as a Michaelis-Menten complex (Fig. 8A) shows that the distance from Ser64Oγ to C9 of FDC is maintained in the 2.0–2.6 Å range during molecular dynamics simulation (MDS). The distances between Lys67Nζ, Tyr150OH, and Lys315Nζ are maintained at the H-bond distance length, and two water molecules are strategically positioned in the active site, at H-bond distances from Lys67, Tyr150, and Ser64. This molecular arrangement suggests a water-mediated acylation mechanism.

For deacylation, both the experimentally determined crystallographic structure of ADC-33/CAZ and the modeled acylated conformation of CAZ were analyzed. We propose that a proton relay occurs from the water to Lys67 via Tyr150 in the formation of the deacylation tetrahedral intermediate, followed by nucleophilic attack of the water at the carbonyl carbon of the former lactam ring. In contrast, studies with ADC-33 and FDC suggest a different mechanism of acylation (Fig. 8). Molecular docking of FDC as an acyl-enzyme complex (Fig. 8B and C) suggests FDC can adopt a different conformation in the ADC-33 active site. In the initial conformation of acyl-FDC (Fig. 8B), multiple water molecules are positioned within hydrogen-bonding distance of FDC. During MDS (Fig. 8C), two waters (W1, W2) are recruited and positioned within hydrogen-bonding distance: (W1) Asn152, N5 of FDC dihydrothiazine ring; (W2) Lys67, Tyr150, N5, and C7 of FDC. A third water molecule (W3) is positioned to coordinate with Tyr150 and Lys315. This suggests that the water molecule could be activated with the help from N5 of FDC and is consistent with a substrate-assisted catalysis mechanism.

## DISCUSSION

To combat the threat of antimicrobial resistance, especially by CR*Ab,* new β-lactam/β-lactamase inhibitor combinations were developed for clinicians to treat multidrug-resistant infections (e.g., sulbactam/durlobactam). Novel agents are in phase III clinical trials (e.g., cefepime/zidebactam) and will soon be ready for evaluation for future clinical use. Other combinations (cefiderocol/xeruborbactam) are also on the horizon.

FDC (Fig. 1G) has been a welcome therapeutic agent to our antimicrobial armamentarium, utilizing an iron transport pathway as a unique mode of entry into the bacterial cell (19). Unfortunately, previous studies demonstrated that certain CR*Ab* isolates had elevated MICs against FDC (≥64 µg/mL) (20). Regrettably, resistance to FDC is emerging due to mutations in *cirA*, the catecholate siderophore receptor-encoding gene (21–24), and expression of β-lactamases (e.g., ADCs, *Pseudomonas* extended resistance [PER], and metallo-β-lactamases) with increased catalytic activity toward FDC (25). Notably, FDC MICs are elevated in clinical *Ab* isolates and isogenic *E. coli* possessing the ADC-33 (5, 6, 26).

Our biochemical characterization of ADC variants, prevalently expressed in CR*Ab*, provides several insights into this problematic group of ESACs that contribute to β-lactam resistance. Initial microbiological studies indicated the possible role of Ω-loop insertions and/or substitutions as a contributing factor to this resistance. The most striking differences were observed between the two groups of variants (Adup versus non-Adup) to confer high-level resistance to expanded-spectrum cephalosporins such as CAZ, ceftolozane, and FDC. Additional characterization of the variants showed remarkable consistency with the microbiology. Stable acyl-adducts were formed between non-Adup ADC-30 and CAZ and FDC that were not observed with Adup ADC-33. Furthermore, measurable hydrolysis of these substrates was shown with both ADC-172 and ADC-33, but not ADC-30. This activity can be attributed to increased destabilization of the enzymes containing insertions and/or mutations in the Ω-loop. Indeed, ADC-33 has a lower *T*_m_ as compared with ADC-30 (55°C versus 60°C). This destabilization suggests mobility or flexibility inherent to the Adup variants that enhances their catalytic abilities with larger cephalosporins.

Addition of residues in the Ω-loop enlarges the Adup active sites. A larger active site is expected to better accommodate bulkier substrates. However, in comparison to Adup variants, binding affinities of CAZ were generally improved in non-Adup variants (*K*_i_ values 2.1 μM–8.7 μM) and were approximately 3- to ~40-fold better than for FDC. A surprising exception was ADC-217 that bound with high affinity to CAZ and FDC at almost equivalent submicromolar *K*_*i*_ values (~0.2 µM). In contrast, Adup variants ADC-33 and -172 bound with slightly higher affinity to FDC than CAZ. Overall, this trend supports an inverse correlation between *K*_*i*_ and hydrolytic turnover of CAZ and, to a lesser extent, FDC. While it is likely that lower *K*_*i*_ values represent tighter binding affinity for CAZ and FDC, it is possible that effects on catalytic rate constants also contribute to the observed differences.

The Adup variants ADC-218 and -219 may represent a subset distinct from the other variants with Ω-loop changes. ADC-218 and -219 do not turn over FDC and are intermediate in their ability to hydrolyze CAZ. However, their low affinity for both CAZ and FDC (*K*_*i*_ >490 µM) would be a viable reason for their lower *k*_cat_/*K*_*M*_ values. Even though ADC-219 contains the same Adup Ω-loop mutations as ADC-33, the susceptibility profile of ADC-219 aligns more closely with non-Adup variants, except for ceftolozane. ADC-219 contains the substitution Gly222Asp. This glycine is conserved in class C β-lactamases, and replacement with an aspartate could alter flexibility in this region, disrupting Tyr221 positioning. In many class C β-lactamase complexes, Tyr221 forms stabilizing stacking interactions with both substrates and inhibitors (4, 27, 28), which could affect the orientation of certain substrates in the active site.

ADC-218 stands out as the ADC variant with the highest levels of resistance to cefotaxime, aztreonam, and CAZ. While classified as an Adup, the location of the alanine duplication is different from the others and contains other variations in that region (Fig. 2). ADC-218 includes Ala200Asp, retains the proline at position 213 (which is arginine in ADC-33 and -172), and includes a Pro219Leu mutation before the duplicated alanine. These differences make ADC-218 unique from the other Adup variants in this study and may allow for active site flexibility or expansion that concomitantly lowers the affinity to some cephalosporins while promoting an environment that supports turnover, leading to increased resistance to cefotaxime, aztreonam, and CAZ. Ongoing crystallographic and molecular dynamics studies will shed light on these structural differences.

Another intriguing observation is the unexpected resistance to cefepime by the non-Adup ADC-217. This resistance is an outlier among the non-Adup variants, yet it tracks with the observed kinetic turnover of cefepime by ADC-217. Interestingly, this variant has the highest affinity for CAZ and FDC, with almost equivalent *K*_*i*_ values (~0.2 µM). In addition, ADC-217 turned over cefepime (*k*_cat_ = 30.2 s^−1^, *K*_*M*_ = 155.2 µM, *k*_cat_/*K*_*M*_ = 0.195 µM^−1^s^−1^), and this maximum variant *k*_cat_/*K*_*M*_ is consistent with the highest MIC against cefepime (16 µg/mL). The strain expressing ADC-172 resulted in the next highest MIC of cefepime (8 µg/mL), and also had the second highest *k*_cat_/*K*_*M*_ of 0.107 µM^−1^s^−1^. Further crystallographic investigation is being performed to understand the mechanism of this increased activity against cefepime.

The X-ray crystal structure of ADC-33 in complex with a β-lactam substrate allows for an investigation into the mechanism of CAZ hydrolysis. Both acyl-enzyme and product forms were fortuitously captured in an enzyme capable of hydrolyzing CAZ. Our analysis supports that substrate-assisted catalysis is the likely mechanism for ADC-33 hydrolysis of CAZ (29, 30). The ADC-33/CAZ complex presented here is consistent with this mechanism. In the acyl-enzyme complex, the carbonyl oxygen is bound productively in the oxyanion hole, and a destabilizing interaction is observed between the acyl-carbonyl oxygen of CAZ and the carbonyl oxygen of Ser318 that may promote efficient deacylation in ADC-33. Additionally, a water molecule (Wat105) is positioned in the active site such that nucleophilic attack at the acyl-carbonyl carbon would be possible. In a substrate-assisted mechanism, either the nitrogen atom (N5) of the former β-lactam ring (4) or the aminothiazole nitrogen (31) has been suggested as the general base that abstracts a proton from a water molecule to activate it for deacylation. In this case, the most likely candidate is the N5 atom of the former lactam ring (~3.4 Å). This proximity is viewed as a long hydrogen bond that appears to increase the propensity for water activation by the substrate. Comparison of this complex was performed with a related acyl-CAZ complex of ADC-7 (Protein Data Bank [PDB] entry 6PWM), a β-lactamase that does not hydrolyze CAZ. Notably, the deacylating water molecule is displaced by 0.7 Å (Fig. 6 and 7), resulting in the presumed deacylating water shifting 0.5 Å closer to its target for deacylation (i.e., the acyl-carbonyl carbon), as well as 0.4 Å closer to the N5 atom of the substrate in the ADC-33 complex.

Tyr150 had previously been suggested as a potential general base in deacylation, which would require the side chain to exist in its deprotonated tyrosinate form. However, several studies point to the likely protonation of the Tyr150 side chain (29, 30, 32). The distances we observe between the deacylating water and the side chain hydroxyl of Tyr150 remain consistent between the ADC-7 and ADC-33 complexes, differing by only ~0.1 Å, which seems to suggest that this single interaction cannot be responsible for the difference in CAZ turnover in ADC-33.

Following deacylation, several changes are observed in the structure that may explain the release of product (Fig. 6). The newly formed carboxylate group has shifted away from the oxyanion hole, no longer in close contact with the carbonyl oxygen of Ser318 but instead clashing with the catalytic serine residue (Ser64). Rotation of the C4′ carboxylate results in the loss of a hydrogen bond with carboxylate recognition residue Asn346. Finally, and perhaps, the largest driver of catalysis may be the unusual conformation of the R1 side chain (Fig. 7). The clash with Tyr221 may favor deacylation and product release in ADC-33. Overall, the lower *T*_*m*_ of ADC-33 suggests an inherent flexibility resulting from both the Ala duplication and Pro213Arg mutation in the Ω-loop.

A certain level of substrate conformational sampling appears to be needed for efficient catalysis and has been achieved with the differences present in the Ω-loop of ADC-33. ADC-172 has the same Ω-loop sequence as ADC-33 and tracks ADC-33 with similar MICs and kinetics with CAZ, although it appears to be slightly improved for FDC, which is concerning. With a substitution in the R2 loop Asn296Lys, this residue is only ~4 Å away from Val262Glu in ADC-217, which demonstrated the highest resistance to cefepime. Interestingly, modeling of the Arg148Gln alteration in ADC-56, another variant with a modest increase in cefepime resistance, suggested that disruption of Arg148 hydrogen bonds (in ADC-30) allows increased flexibility in the H-10 helix within the R2 loop and possibly facilitates turnover of cefepime by ADC-56 (7). Arg148 is adjacent to two active site residues (Tyr150 and Asn152), and any possible mutations in that R2 loop region could affect Arg148 interactions, thereby facilitating enhanced binding and turnover of specific substrates, like ADC-172 and ADC-217 with cefepime.

**Fig 9:**
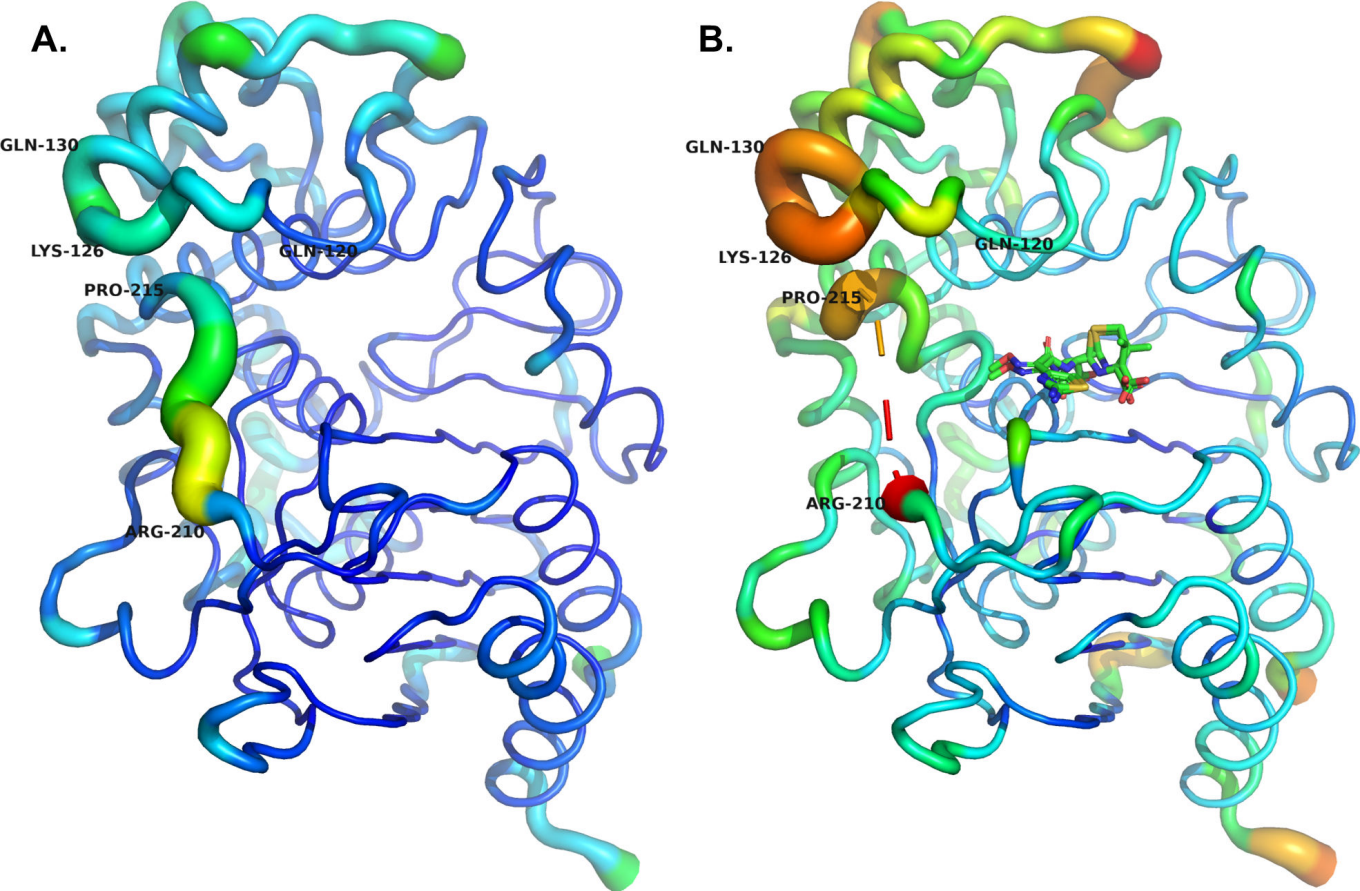
Analysis of B-factors for ADC-33 in the (**A**) apo (PDB 8FQN) form, and (**B**) ceftazidime complex (PDB 9EHY). In the complex, the Ω-loop is disordered with no electron density for residues 211–214, depicted as a dashed line. The range in B-factors is indicated with a color scale (where low to high B’s range from blue to red), as well as tube size (where low to high B’s range from small to large).

How do these complexes inform our understanding of ADC-33 hydrolysis of CAZ? Might this apply to all the variants containing insertions or mutations in the Ω-loop region, and what does this tell us about drug design? The Ω-loop has been identified as a “hot spot” for mutations that result in gaining expanded-spectrum activity against a wider range of β-lactam antibiotics. Previous studies with classes A, C, and D β-lactamases report increased flexibility of this loop and others surrounding the catalytic center, which results in a larger active site. This expansion may permit hydrolysis of bulkier molecules, like the expanded-spectrum cephalosporins, via either improved substrate access or product release (33–37). In the class A KPC-2 with the Asp179Tyr substitution, MDS indicated significantly more flexibility in the CAZ-liganded enzyme when compared with apoKPC-2 (33). Analysis of the ADC-33/CAZ complex is consistent with these previous studies. CAZ binding in ADC-33 appears to induce a level of flexibility in the Ω-loop that resonates to the loop above it (residues 123–131; Fig. 9), like KPC-2 Asp179Tyr. In our complex, a portion of the Ω-loop (residues 212–215) could not be modeled due to weak or absent electron density. Overall average B-factors of apoADC-33 (8FQN) are much lower (protein atoms 18.79 Å^2^) than those of the ADC-33/CAZ complex (33.98 Å^2^). Additional hallmarks of flexibility are also suggestive in the complex: capture of both an acylated and product form of CAZ at reduced occupancies, weak electron density for the dimethylcarboxylate group of the R1 side chain. Perhaps this is unsurprising given that ADC-33/CAZ reveals a static picture of an enzyme active site undergoing hydrolysis of CAZ, and the substrate may be sampling conformations to achieve one that is competent for catalysis. Overall, loop flexibility, displacement of the deacylating water, and destabilization of the acyl-enzyme intermediate appear to favor hydrolysis of CAZ and product release in ADC-33.

While the ADC Adup variants show an overall trend of increasing resistance to larger cephalosporins, cefiderocol is largely spared from the gain in resistance by the Adup variants, as all tested ADC variant isolates are in the susceptible range (≤4 mg/L) (Table 1) (38). ADC-33, -172, -218, and non-Adup ADC-217 show increased MICs against cefepime over other non-Adup variants, but the overall MICs still demonstrate susceptibility to cefepime, except for ADC-217 (16 µg/mL; intermediate range). CAZ and ceftolozane also have bulkier R2 groups (Fig. 2). What structure and/or chemistry would allow FDC and cefepime to retain effectiveness against the ADCs? Both FDC and cefepime contain an aliphatic pyrrolidinium ring, whereas CAZ and ceftolozane contain the aromatic heterocycles pyridine and pyrazole, respectively. During acylation, these heterocycles act as neutral leaving groups. Thus, it is likely that the lower pK_a_ of the pyridines and pyrazoles (pK_a_ 5 for the conjugated acids) (39) versus the pyrrolidines (pK_a_ 11) (40) may lead to faster acylation of CAZ or ceftolozane in comparison to FDC and cefepime.

Pairing novel inhibitors with current cephalosporins may be an extremely effective strategy to prevent the clinical emergence of these ESACs. We previously showed MB076 can be an inhibitor of ADC-33 (5). This leads us to postulate that a boronate with a catechol-like structure to increase bacterial cell penetration may be a useful agent against CR*Ab*. Efforts are underway to explore this possibility.

## MATERIALS AND METHODS

### Antimicrobial susceptibility testing

Susceptibility testing to standard antibiotics was performed by broth microdilution according to 2024 Clinical and Laboratory Standards Institute (CLSI) guidelines (38). MICs for FDC were done in iron-depleted cation-adjusted Mueller-Hinton (MH) broth according to CLSI methods. All MICs were interpreted according to the 2024 CLSI guidelines (38). MICs are done in triplicate, and the mode MIC value is reported.

#### Plasmid constructs in pBCSK− for MIC determinations

*bla*_ADC-7_ pBCSK− was cloned and expressed in *E. coli* DH10B cells as previously described for MIC determinations (3). The pBCSK-non-inducible phagemid vector has a chloramphenicol resistance gene used to aid selection. All other *bla*_ADC_ variants were synthesized by GenScript according to the *bla*_ADC-7_ pBCSK− strategy, cloning into the XbaI/BamHI sites of the pBCSK− vector (5).

### Expression and purification of ADC variants

ADC-7 β-lactamase was expressed as previously described and purified using cation exchange chromatography (4, 6). The expression plasmids for all other ADC variants were constructed in pET28a vectors by GenScript. The purification and quantification of all ADCs were performed as previously described (5).

### Kinetic characterization of ADC variants

Steady-state kinetic parameters were determined by combining pure enzyme with antibiotic substrates in 50 mM NaH_2_PO_4_, pH 7.4, at room temperature. Changes in absorbance were measured on the Cary 60 UV−Vis spectrophotometer (Agilent Technologies) and converted to velocity using the change in extinction coefficient specific to nitrocefin (NCF) (ε_482_ = 17,400 M^−1^·cm^−1^), CAZ (ε_260_ 8,660 M^−1^·cm^−1^), and cefiderocol (ε_259_ 9,430 M^−1^·cm^−1^). The concentration of enzyme ranged from 0.1 to 1.0 µM, and all kinetic experiments were performed at least in triplicate. Initial velocities were fitted to the Michaelis-Menten equation, yielding *k*_cat_ and *K*_*M*_ values. The standard error for *k*_cat_/*K*_*M*_ values was generated by factoring in the larger of the proportional standard errors for the individual *k*_cat_ value and the *K*_*M*_ value. For ADC/substrate combinations in which the *K*_M_ was too large to accurately determine both *k*_cat_ and *K*_*M*_ values, the initial velocities at *k*_cat_*/K*_*M*_ values were calculated from linear fits of *v_o_* in low [S] ranges (<0.08 * *K*_*M*_).

For inhibition kinetics, utilizing NCF as a colorimetric substrate of ADCs, the inhibition constant (*K*_*i*_) of CAZ and FDC with ADCs was determined using competition kinetics as previously described (5). After a 3 min pre-incubation of the enzyme (2 nM) with increasing concentration of the inhibitor, measurements of the initial velocities were performed with the addition of 100–200 µM NCF (twofold of the experimentally determined *K*_*M*_ value). To determine the average velocities (*v*_0_), data from three experiments were fit to the followingequation:


$$v_{0}=v_{u\;}-\left\{\frac{v_{u}\left[I\right]}{{IC}_{50}+\left[I\right]}\right\}$$

where *v_u_* represents the NCF uninhibited velocity and IC_50_ represents the inhibitor concentration that results in a 50% reduction of *v_u_*. The *K*_*i*_ value was corrected for the NCF affinity (*K*_*M*_ values for each ADC) with the Cheng-Prusoff (5) equation:


$$K_{i}={IC}_{50}/\left(1+\frac{\left[NCF\right]}{K_{mNCF}}\right)$$

The standard error for *K*_*i*_ values was generated by factoring in the larger of the proportional standard errors for the individual IC_50_ value and the *K*_*M* -NCF_ value.

### Thermal denaturation and stability

For thermal denaturation, protein samples were monitored for helical content by circular dichroism in the far-UV region at 210 and 220 nm using a Jasco J-815 spectrometer (Easton, MD) with a Peltier-effect temperature controller, as previously described (41). Enzyme concentrations were 12 µM in phosphate-buffered saline (PBS) pH 7.4. The thermodynamic stability of apoADC-30 and ADC-33, and with 1 mM FDC and CAZ, was determined from equilibrium unfolding curves. With the assumption of a reversible two-state transition, we determined the *T*_*m*_ at the midpoint of equilibrium folding. Thermal denaturation experiments were done in duplicate under the same conditions, with each data point representing the average of three successive scans, and the *T*_*m*_ values are consistent with ±1°C variation.

### Crystallization and X-ray crystal structure determination of ADC variants

ADC-33 crystals were grown via hanging drop vapor diffusion at room temperature as previously described (41, 42). The ADC-33/CAZ complex was obtained by harvesting a preformed crystal, soaking it in crystallization buffer containing CAZ at 70 mM for 55 min, then flash cooling it in liquid nitrogen.

Data were measured from a single crystal at the Advanced Photon Source at Argonne National Laboratory (LS-CAT 21ID-F). Diffraction data were processed with autoPROC (43), and additional processing of the structure factors was performed using STARANISO (44). Structures were determined by molecular replacement with Phaser (45) using as a starting model the structure of apoADC-33 (8FQN) with all Ω-loop residues, waters, inhibitor, and ion atoms removed. The model was refined using Phenix (46, 47), followed by model building in Coot (48). Polder omit maps were calculated with Phenix by omitting the ligand and using a 3.0 Å solvent exclusion radius (49).

### Molecular modeling and docking

Coordinates of ADC-33 (8FQN) were used to construct Michaelis-Menten and acyl-enzyme models of FDC and CAZ, using BIOVIA Discovery Studio 2020 Client (Dassault Systèmes). Structures of CAZ and FDC were constructed using Fragment Builder tools and minimized using a Standard Dynamics Cascade protocol of DS 2020. Intact and hydrolyzed compounds were docked into the active site of ADC-33 using the Flexible Docking module of DS 2020. After docking, the most favorable pose was chosen based on the minimum distance between the FDC carbonyl C8 and Ser64. The complex was created with a covalent bond introduced between Ser64 and C8 of the hydrolyzed β-lactam. To explore possible conformational changes of the β-lactamase-ligand complex, MDS was conducted. Each stage of MDS was performed for 150 ps.

### Mass spectrometry

β-Lactamase (5 µg) was incubated with substrate at a molar ratio of 1:25 (FDC, CAZ, meropenem [MEM]) in sterile 10 mM PBS, pH 7.4, for a total reaction volume of 20 µL (*t* = 30 s, 1 min, 15 min, 30 min, 1 h, 2 h, and 24 h). Reactions were quenched with 1% acetonitrile and 0.1% formic acid. Samples were analyzed using Q-TOF Waters Synapt-G2-Si and a Waters Acquity ultrapressure liquid chromatography BEH C18 column (1.7 µm pore size; 2.1 × 50 mm). MassLynx v.4.1 was used to deconvolute protein peaks. The tune settings for each data run were as follows: capillary voltage at 3.5 kV, sampling cone at 35, source offset at 35, source temperature 100°C, desolvation temperature 500°C, cone gas at 100 L/h, desolvation gas at 800 L/h, and nebulizer bar at 6.0. Mobile phase A was 0.1% formic acid-water. Mobile phase B was 0.1% formic acid-acetonitrile. The mass accuracy for this system is ±5 Da. Mass spectrometry experiments were performed once over a range of time points.

## Data Availability

Coordinates and structure factors are deposited with the Protein Data Bank as 9EHY.

## SUPPLEMENTAL MATERIAL

Supplemental material mbio.00287-25-s0001.pdf Supplemental table and figures.

ASM does not own the copyrights to Supplemental Material that may be linked to, or accessed through, an article. The authors have granted ASM a non-exclusive, world-wide license to publish the Supplemental Material files. Please contact the corresponding author directly for reuse.
