# Supplementary material for: Resistance to oxyimino-cephalosporins conferred by an alternative mechanism of hydrolysis by the Acinetobacter-derived cephalosporinase-33 (ADC-33), a class C β-lactamase present in carbapenem-resistant Acinetobacter baumannii (CRAb)
Source: mBio. 2025 May 16;16(6):e00287-25. doi: 10.1128/mbio.00287-25 (PMC12153338; doi:10.1128/mbio.00287-25)
Supplement: Supplemental material — Supplemental table and figures. [file mbio.00287-25-s0001.pdf]

Resistance to oxyimino-cephalosporins conferred by an alternative mechanism of hydrolysis by the *Acinetobacter*-Derived Cephalosporinase-33 (ADC-33), a class C β-lactamase present in carbapenem-resistant *Acinetobacter baumannii* (CR*Ab*)

Running title: Mechanism of ceftazidime hydrolysis by ADC-33

Rachel A. Powers[a#*], Bradley J. Wallar[a#*], Hannah R. Jarvis[a], Zoe X. Ziegler[a], Cynthia M. June[a], Christopher R. Bethel[b], Andrea M. Hujer[c], Magdalena A. Taracila[b,c], Susan D. Rudin[b,c], Kristine M. Hujer[c], Fabio Prati[d], Emilia Caselli[d], Robert A. Bonomo[b,c,e,f,g*]

[a] Department of Chemistry, Grand Valley State University, Allendale, MI, USA;

[b] Research Service, Louis Stokes Cleveland Department of Veterans Affairs Medical Center, Cleveland, OH, USA;

[c] Department of Medicine, Case Western Reserve University School of Medicine, Cleveland, OH, USA;

[d] Department of Life Sciences, University of Modena and Reggio Emilia, Modena, Italy;

[e] Clinician Scientist Investigator, Louis Stokes Cleveland Department of Veterans Affairs Medical Center, Cleveland, OH, USA;

[f] Departments of Pharmacology, Molecular Biology and Microbiology, Biochemistry, and Proteomics and Bioinformatics, Case Western Reserve University School of Medicine, Cleveland, OH, USA;

[g] CWRU-Cleveland VAMC Center for Antimicrobial Resistance and Epidemiology (Case VA CARES) Cleveland, OH, USA.

* Corresponding authors, Robert.bonomo@va.gov; powersra@gvsu.edu; wallarb@gvsu.edu

# These authors contributed equally to the manuscript

Supplement Table S1: Crystallographic statistics of ADC-33/ceftazidime complex

	ADC-33/ceftazidime
Cell constants (Å;°)	*a=43.13, b=84.06* *c*=205.60; $\alpha=\beta=\gamma=90$
Space group	$P\ 2_1 2_1 2_1$
Resolution (Å)	102.80–1.57 (1.72–1.57)[a]
Unique reflections	79,527 (3,975)
Total reflections	629,270 (35,460)
R_{merge} (%)	6.9 (98.4)
Rpim (%) (all I + I-)	2.6 (34.9)
CC(1/2)	0.999 (0.723)
Completeness (%)[b]	93.5 (58.8)
Mean(I)/σ(I)	15.0 (1.7)
Resolution for refinement (Å)	43.85–1.57
No. of protein residues	704
No. of waters	405
RMSD bond lengths (Å)	0.008
RMSD bond angles (°)	0.889
R-factor (%)	19.2
R_{free} (%)[c]	22.0
Average B factor, protein (Å^2)	35.34
Average B factor, ligand (Å^2)	46.52
Average B factor, waters (Å^2)	36.62

[a] Highest resolution shell indicated in parentheses. Subsequent values in parentheses are for that shell.
[b] Fraction of theoretically possible reflections.
[c] R_{free} was calculated with 5% of reflections set aside randomly.

Supplement Figure S1

Qualitative immunoblot analysis of ADC variant expression levels. Immunoblotting was performed as previously described (1), with the exception that all samples were probed with 1 μg/ml of the anti-ADC-7 polyclonal rabbit antibody. Purified ADC-7 β-lactamase was diluted in a range of concentrations and used to load the following amounts: 500, 300, 100, and 50 ng. Detection of ADC β-lactamases was determined by chemiluminescence, and the anti-DNase K antibody was used as a loading control probe. Epitope mapping of the ADC-7 β-lactamase demonstrated two major antibody reactive epitopes that occur within the Ω loop. They are amino acids 196-210: MQNYAFGYNQENQPIR and 213-225: PGPLDAPAYGVKS (SANC numbering, data not shown). Varying this antigenic region, as is the case with some of the ADC variants in this study, can affect binding of the anti-ADC antibody. Therefore, we present the immunoblot results to demonstrate protein production of all variants. However, quantification of the amounts cannot be determined for all ADC variants.

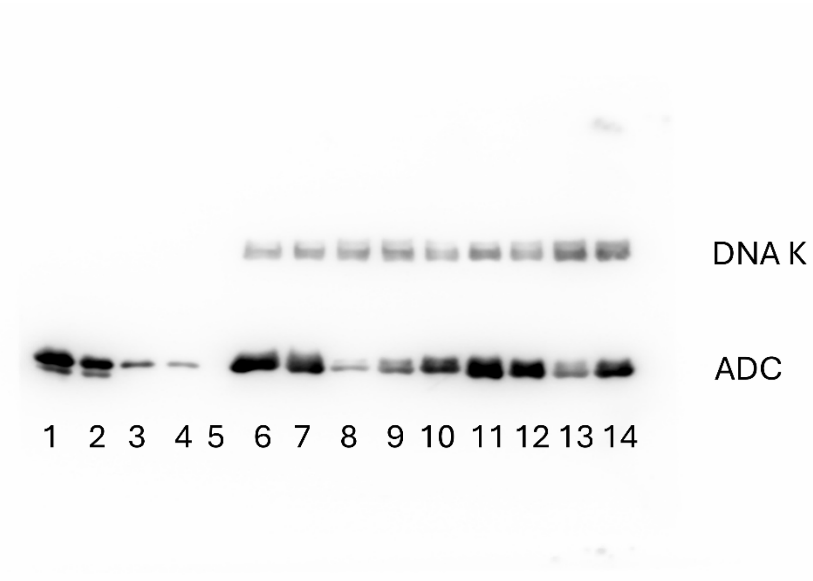

Lane
1 500 ng ADC-7
2 300 ng ADC-7
3 100 ng ADC-7
4 50 ng ADC-7
5 Protein Markers
6 ADC-7
7 ADC-30
8 ADC-73
9 ADC-143
10 ADC-217
11 ADC-33
12 ADC-172
13 ADC-218
14 ADC-219

Supplement Figure S2

Michaelis-Menten plots for the turnover of ceftazidime (CAZ) and cefiderocol (FDC) by ADC-33 and ADC-172. Steady-state kinetic parameters were determined by combining pure enzyme with antibiotic substrates in 50 mM NaH_2PO_4, pH 7.4 at room temperature. Changes in absorbance were measured on the Cary 60 UV−Vis spectrophotometer (Agilent Technologies) and converted to velocity using the change in extinction coefficient specific to CAZ (ε_{260} = 8660 $M^{-1}\cdot cm^{-1}$) or FDC (ε_{259} = 9430 $M^{-1}\cdot cm^{-1}$). The concentration of enzyme for ADC-172/CAZ turnover was 0.1 µM, while the rest of the experiments utilized 1 µM enzyme. All experiments were performed in triplicate. Initial velocities were fitted to the Michaelis-Menten equation yielding k_{cat} and K_M values.

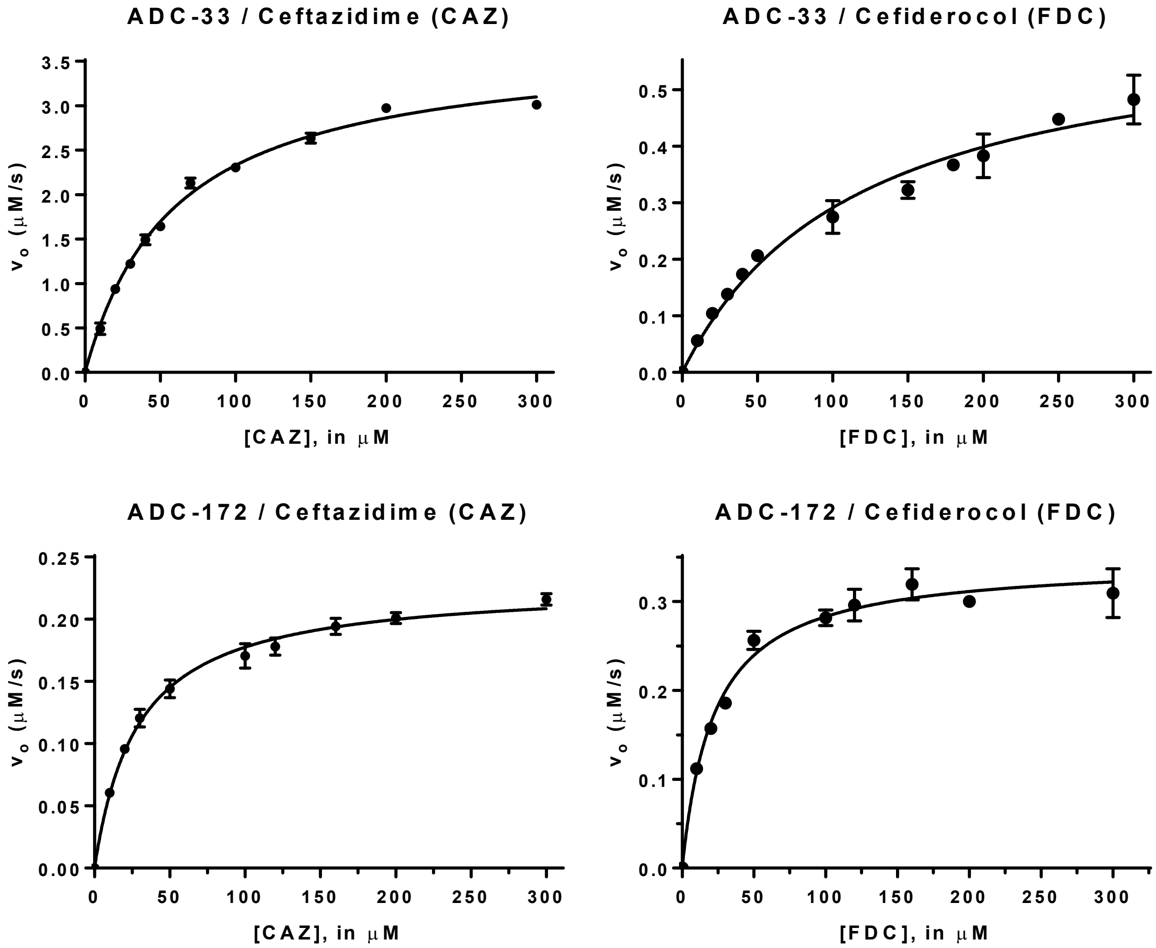

Supplement Figure S3

Stereoview of Polder omit maps in the active site of ADC-33. The Polder omit map was calculated with Ser64 and ceftazidime (CAZ and A1BIM) excluded. The omit map is shown as a gray cage surrounding the excluded residues and is contoured at 4.0 σ. Carbon atoms of CAZ are colored cyan, and those of A1BIM are dark teal. Polder omit maps were calculated on the final model and confirm the conformations of CAZ in each active site. Occupancies for CAZ in the B monomer refined to 0.42 (acyl-CAZ) and 0.40 (product A1BIM), and in the A monomer, the occupancy of the product refined to 0.70. Since the occupancy for CAZ is not 100%, waters (Wat105 in B and Wat416 and Wat418 in A) were added to remaining difference electron density maps (3 σ). Given that overall B factors for protein (B 33.98 $Å^2$ vs. A 36.62 $Å^2$) and ligand atoms (B CAZ 43.59 $Å^2$, A1BIM 43.36 $Å^2$ vs. A A1BIM 52.88 $Å^2$) are lower in the B monomer, as well as that both acylated and product forms are present, this monomer is presented as representative of both in analysis and figures.

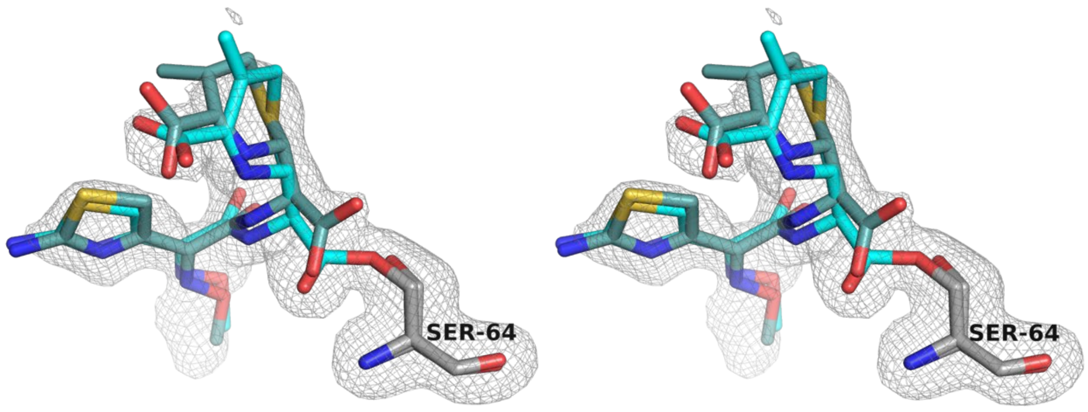

Supplement Figure S4

Connolly surface representations of ADC-33 with (**A.)** CAZ (represented as cyan sticks) and (**B.**) FDC (yellow sticks) docked as Michaelis-Menten complexes into the active site. The modeled Michaelis-Menten form of FDC can be accommodated in the active site, although the molecule is more restricted than CAZ due to the larger catechol moiety in the R2 group.

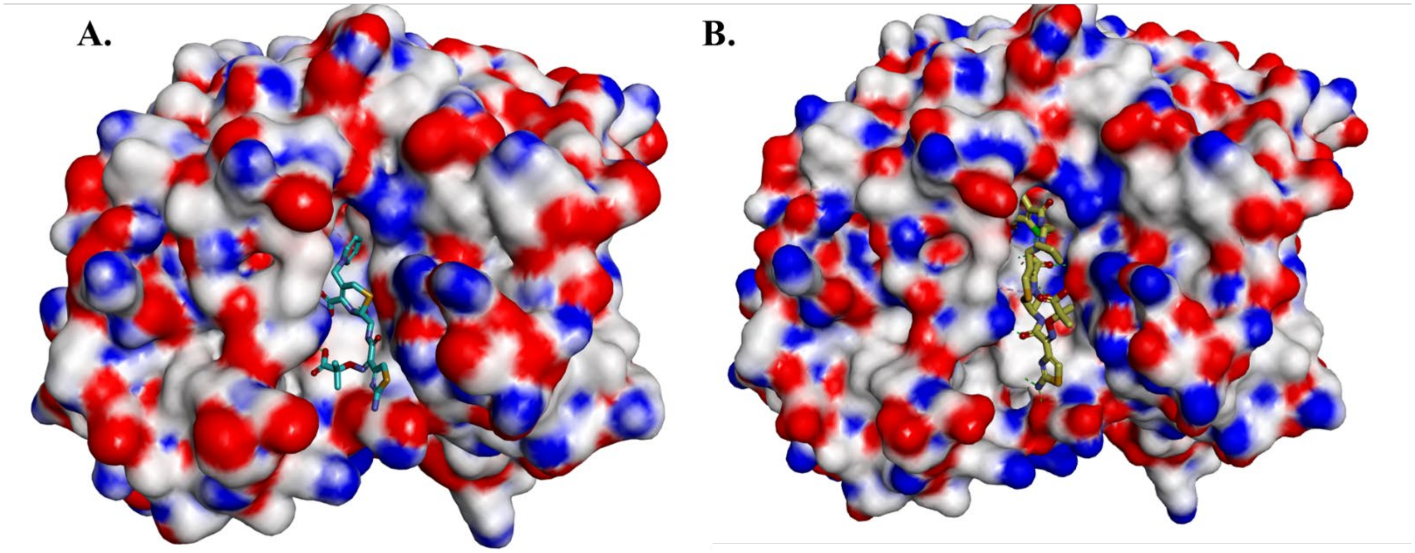

Reference

1. Hujer AM, Page MG, Helfand MS, Yeiser B, Bonomo RA. 2002. Development of a sensitive and specific enzyme-linked immunosorbent assay for detecting and quantifying CMY-2 and SHV beta-lactamases. J Clin Microbiol 40:1947-57.
